# Bone as a Possible Target of Chemical Toxicity of Natural Uranium in Drinking Water

**DOI:** 10.1289/ehp.7475

**Published:** 2004-09-30

**Authors:** Päivi Kurttio, Hannu Komulainen, Aila Leino, Laina Salonen, Anssi Auvinen, Heikki Saha

**Affiliations:** ^1^STUK–Radiation and Nuclear Safety Authority, Research and Environmental Surveillance, Helsinki, Finland; ^2^Division of Environmental Health, National Public Health Institute, Kuopio, Finland; ^3^Department of Clinical Chemistry, Turku University Hospital, Turku, Finland; ^4^School of Public Health, University of Tampere, Tampere, Finland; ^5^Department of Internal Medicine, Tampere University Hospital, Tampere, Finland

**Keywords:** bone, bone turnover markers, CTx, drinking water, osteocalcin, P1NP, uranium

## Abstract

Uranium accumulates in bone, affects bone metabolism in laboratory animals, and when ingested in drinking water increases urinary excretion of calcium and phosphate, important components in the bone structure. However, little is known about bone effects of ingested natural uranium in humans. We studied 146 men and 142 women 26–83 years of age who for an average of 13 years had used drinking water originating from wells drilled in bedrock, in areas with naturally high uranium content. Biochemical indicators of bone formation were serum osteocalcin and amino-terminal propeptide of type I procollagen, and a marker for bone resorption was serum type I collagen carboxy-terminal telopeptide (CTx). The primary measure of uranium exposure was uranium concentration in drinking water, with additional information on uranium intake and uranium concentration in urine. The data were analyzed separately for men and women with robust regression (which suppresses contributions of potential influential observations) models with adjustment for age, smoking, and estrogen use. The median uranium concentration in drinking water was 27 μg/L (interquartile range, 6–116 μg/L). The median of daily uranium intake was 36 μg (7–207 μg) and of cumulative intake 0.12 g (0.02–0.66 g). There was some suggestion that elevation of CTx (*p* = 0.05) as well as osteocalcin (*p* = 0.19) could be associated with increased uranium exposure (uranium in water and intakes) in men, but no similar relationship was found in women. Accordingly, bone may be a target of chemical toxicity of uranium in humans, and more detailed evaluation of bone effects of natural uranium is warranted.

Increased uranium levels in groundwater are associated with uranium-rich ores and high solubility of uranium under oxidizing conditions in soft and bicarbonate-rich waters (Salonen 1994). Consequently, exceptionally high uranium concentrations have been found in private drilled wells located mostly in the southern part of Finland (Salonen and Huikuri 2002). We identified earlier a cohort of people who live in that area and use drilled wells for drinking water (Kurttio et al. 2002).

In long-term exposure, uranium accumulates in the bone and kidneys (Leggett and Pellmar 2003; Pellmar et al. 1999). The kidney has been considered the main target organ of chemical toxicity of uranium in humans, but effects in other tissues or organs remain poorly known. The intake of natural uranium through drinking water is associated with kidney function, particularly increased fractional excretion of calcium and phosphate in urine (Kurttio et al. 2002). The implications of the accumulation of natural uranium in the bone in humans are not known. The early distribution of uranium in the skeleton is similar to that of calcium (Leggett 1994). Uranium is assumed to deposit on the bone surface, and the uranyl ion (UO_2_^2+^) is assumed to be exchanged with calcium ions at the surfaces of bone mineral crystals but not to participate in crystal formation (Leggett 1994). Gradually, uranium is redistributed in the bone and other tissues. The current biokinetic model of the International Commission on Radiological Protection suggests three compartments for uranium in human bone: bone surface, exchangeable bone volume, and nonexchangeable bone volume (Leggett 1994). It also suggests that uranium leaves bone surfaces more slowly than does calcium and that the removal from the nonexchangeable bone compartment may occur but with the rate of bone turnover.

The resemblance of uranium metabolism to that of calcium in bone enables the effects of uranium on bone. Indeed, uranium administration in rats is known to affect the bone. Acute (Guglielmotti et al. 1984) or continuous (Diaz Sylvester et al. 2002) exposure to uranium may lead to decreased bone formation rate and also increased bone resorption (Ubios et al. 1991) in rats.

The aim of this study was to assess whether uranium intake through drinking water affects the biochemical markers of bone turnover in humans. The present study extends our previous study, which suggested that uranium exposure is associated with altered proximal renal tubular function (Kurttio et al. 2002). To our knowledge, this is the first report on the possible effects of ingested natural uranium on bone in humans.

## Materials and Methods

### Study population.

The source population was identified from the drinking water database of STUK–Radiation and Nuclear Safety Authority, with radionuclide analyses of more than 5,000 drilled wells. This study was limited to southern Finland, where uranium concentrations are highest. The study population was a subpopulation of our previous study on effects of natural uranium on kidney function, with a more detailed description published earlier (Kurttio et al. 2002). The first questionnaire was mailed to 798 households. Based on the first questionnaire, 436 persons were selected, with a maximum of two persons per household where a drilled well had been used for drinking water at least for the previous year (median duration of use, 11 years). The second questionnaire was used to collect information on residential history and use of drilled well water and its daily consumption, use of other beverages, smoking history, education, occupation, disease history, and use of medication and herbal products. Seventy-eight percent of the persons who received the second questionnaire agreed to participate in the study (samples were received from these persons). Further information on, for example, bone fracture history and information on menopause and on physical activity was also collected (67% replied to this third questionnaire). We do not have information on type or date of the bone fractures.

Subjects were excluded if they were < 25 years of age (*n* = 11); had diabetes mellitus (*n* = 4); reported long-term use of glucocorticoids (*n* = 11), thiazide diuretics (*n* = 7), methotrexate (*n* = 1), or sodium aurothiomalate (*n* = 1); were currently pregnant (*n* = 4); or used effective equipment for removing uranium from well water (*n* = 4). The final study population consisted of 288 persons from 179 households. Most of the study persons had never smoked, and their average body mass index was 25 kg/m^2^ (Tables 1 and 2). Twenty-six women used estrogen (oral contraceptives or hormonal replacement therapy) regularly during the previous year, and the women had had two deliveries on average. None of the subjects reported hyperparathyroidism.

The study protocol was approved by the National Public Health Institute Standing Committee on Ethics (project 8/030399).

### Sample collection and preparation.

The water, urine, and nonfasting blood samples were collected between 14 September and 1 December 1999. The samples were collected at a time when the study persons had consumed water from the drilled well throughout the previous week. Samples were not taken unless at least 1 week had elapsed since an acute infection. The study persons brought the water and urine samples collected overnight to the laboratory in the morning. At the same visit, blood samples were taken (77% of the samples were taken before 1100 hr). In addition, body weight and height were measured in a standardized fashion. The water and overnight urine samples for uranium analyses were conserved with concentrated HNO_3_. Water samples were stored at room temperature but serum and urine samples frozen at −20°C until analyzed.

### Uranium exposure assessment.

Uranium in drinking water and urine were analyzed blind with inductively coupled plasma mass spectrometry. The analysis and quality control procedure are described by Kurttio et al. (2002). The primary measure of uranium exposure was uranium concentration in drinking water (micrograms per liter). In addition, we measured daily intake of uranium from drinking water (volume used × concentration, micrograms), cumulative intake from drinking water (daily intake × duration of the water consumption, grams), and uranium concentration in urine (micrograms per liter or micrograms per millimole creatinine). The exposure variables were highly correlated with each other (Table 3).

### Outcome variables.

Serum osteocalcin and amino-terminal propeptide of type I procollagen (P1NP) were used as indicators of bone formation, reflecting different stages of osteoblast differentiation. We analyzed osteocalcin using an immunoradiometric assay, which measures human osteocalcin (1–49) and human osteocalcin peptide (1–43) (ELSA-OSTEO; CIS Bio International, Gifsur-Yvette, France). At the 15-μg/L level, the intra- and interassay variations were 2.0 and 3.1%, respectively. P1NP was analyzed with a commercial radio-immunoassay (Procollagen Intact P1NP; Orion Diagnostica, Oulunsalo, Finland); intra- and interassay variations at the 40-μg/L level were 2.0 and 4.9%, respectively.

Serum type I collagen carboxy-terminal telopeptide (CTx) was used as an indicator of bone resorption. CTx was analyzed with an enzyme immunoassay (Serum CrossLaps One Step ELISA; Osteometer Biotech, Herlev, Denmark); intra- and interassay variations at the 2.4-nmol/L level were 6.4 and 7.2%, respectively.

In men, the correlation between the log-transformed osteocalcin and P1NP was 0.70; between osteocalcin and CTx, 0.38; and between P1NP and CTx, 0.32. In women, the correlations were 0.63, 0.46, and 0.36, respectively.

Urine calcium was measured with atomic absorption spectrophotometry (EFOX 5053; Eppendorf, Hamburg, Germany) (detection limit, 0.1 mmol/L). Urine phosphate was measured based on a colored complex with ammonium molybdate (Konelab 60I; Konelab Co., Espoo, Finland) (detection limit, 2.0 mmol/L). Urinary excretions of calcium and phosphate (millimoles per hour) were calculated from the volume of urine divided by the overnight collection time. The mean ± SD excretion of urine was 79 ± 36 mL/hr in men and 78 ± 37 mL/hr in women.

### Statistics.

For all the parameters determined, the observations below the detection limits were recorded as half of the detection limit. An analysis of the residuals indicated that they were not normally distributed and that some of the observations were highly influential. Therefore, the robust regression method using iteratively reweighted least squares (Huber and Tukey bi-square weight function) with rreg routine in Stata/SE 8.1 for Windows (Stata Corp., College Station, TX, USA) was used. Robust regression assigns a weight to each observation, with lower weights given to possible influential observations. Some results from the conventional linear regression are also given in “Results.”

The analyses were performed separately for men and women. For men, markers of bone metabolism levels were modeled using linear and quadratic terms for age and a variable for current smoking. The model used for women included a categorical age term [< 45 (reference), 45–55, 55–65, and ≥65 years], with additional variables for recent regular estrogen use and current smoking.

Algebraically these are as follows:


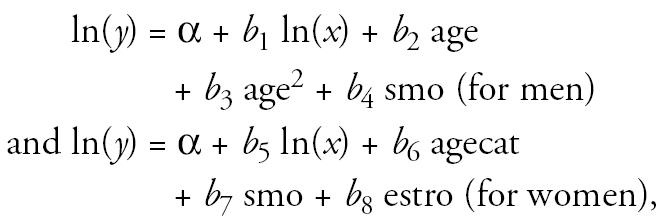


in which *y* is the indicator of bone metabolism, α is a constant, *b* is the regression coefficient, *x* is continuous uranium exposure, agecat is age category (45–55, 55–65, and ≥65 years), smo is current smoking status, and estro is use of estrogens, the last two being binary indicator (dummy) variables. *R*^2^ and *p*-values of the models are described in Table 4.

Analyses were also carried out using the above-mentioned models with log-transformed urinary calcium or phosphate excretions as explanatory variables.

## Results

### Background.

**Figure 1:**
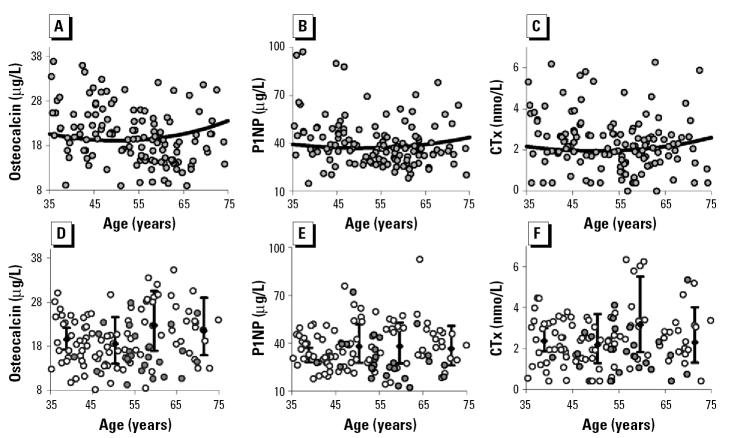
Background levels of the markers of bone turnover in men (*A–C*) and women (*D–F*). The lines represent the estimates from the robust regression models described in the text. In *A–C*, curvature lines and *p*-values represent the estimates of squared age variable. All *p*-values for associations between the linear age variable and all outcome variables were < 0.01. In *D–F*, open circles represent women who do not use estrogen, and shaded circles, women who use estrogen. Error bars are 95% confidence intervals of age groups. *p*-Values are given for the of 55–65-year age group compared with the < 45-year age group and for the estrogen users compared with nonusers (estro): (*A*) *p* = 0.06; (*B*) *p* = 0.13; (*C*) *p* = 0.03; (*D*) *p* = 0.08 (55–65 years), *p* = 0.005 (estro); (*E*) *p* = 0.09 (55–65 years), *p* < 0.001 (estro); (*F*) *p* = 0.07 (55–65 years), *p* = 0.001 (estro).

For men, the levels of osteocalcin, P1NP, and CTx tended to decrease with age until about 60 years of age, after which bone turnover appeared to increase gradually but age accounts for a relatively small proportion of the variation in the bone turnover measurements (Figure 1). All *p*-values were < 0.01 for associations between the linear age variable and all outcome variables. In men, current smoking was associated with decreased levels of osteocalcin (*p* = 0.01), P1NP (*p* = 0.11), and CTx (*p* = 0.03).

For women, the levels of osteocalcin, P1NP, and CTx were highest in the age group of 55–65 years, but the differences between the age groups were not statistically significant (Figure 1). Smoking in women was not statistically significantly associated with any marker of bone turnover. Estrogen use was associated with significantly decreased levels of osteocalcin, P1NP, and CTx (Figure 1).

### Uranium exposure.

The uranium concentration in water varied from 0.001 to 1,920 μg/L (Table 2), with 27% of the concentrations > 100 μg/L and 59% > 15 μg/L. The median daily intake of uranium from drinking water was 36 μg, and the cumulative intake was 120 mg. The median annual committed equivalent radiation dose of bone surfaces was 0.36 mSv/year (maximum, 41 mSv/year), based on the uranium intake and the average uranium isotope activity ratios measured in Finnish drilled well waters (^234^U:^238^U = 2) and dose conversion factors (^234^U, 7.4 × 10^−7^ Sv/Bq; ^238^U, 7.1 × 10^−7^ Sv/Bq) (International Commission on Radiological Protection 1994).

**Figure 2:**
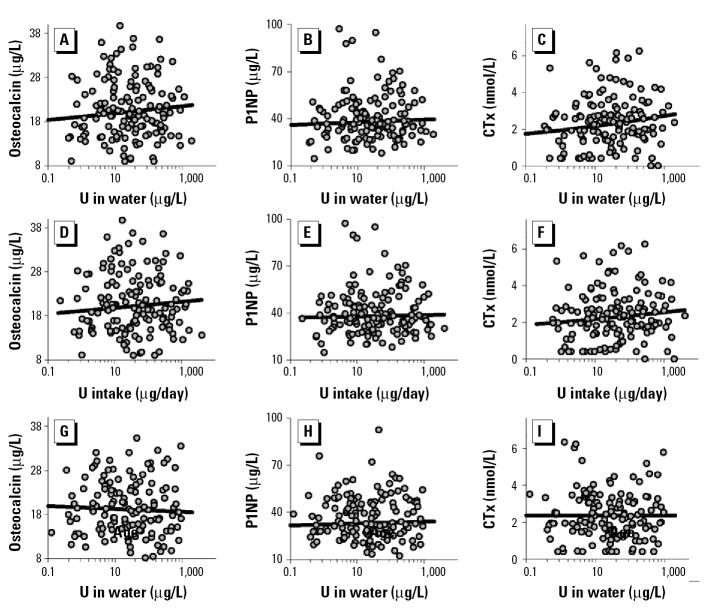
Associations between biochemical markers of bone turnover and uranium (U) exposure expressed as concentration in drinking water or as daily intake in men (*A–F*) and women (*G–I*). Regression lines and *p*-values were taken from the robust regression models. (*A*) *p* = 0.19; (*B*) *p* = 0.46; (*C*) *p* = 0.05; (*D*) *p* = 0.23; (*E*) *p* = 0.71; (*F*) *p* = 0.16; (*G*) *p* = 0.58; (*H*) *p* = 0.60; (*I*) *p* > 0.99.

In men, uranium exposure was associated with elevated CTx levels (Figure 2) with the *p*-values in the robust regression 0.05 for uranium in water, 0.16 for daily intake, and 0.16 for cumulative intake. The corresponding *p*-values in conventional linear regression analyses were 0.01, 0.02, and 0.03. There was an indication of an association between increased levels of osteocalcin and uranium concentrations in drinking water (*p* = 0.19; *p*-value in the conventional linear regression was 0.04). Levels of P1NP were not associated with uranium exposure. Uranium concentrations in urine expressed as micrograms per liter or micrograms per millimole creatinine were not associated with the markers of bone turnover.

Increased urinary excretion of calcium tended to be associated with increased CTx levels (*p*-value from the robust regression was 0.10), and some indication was found for increased urinary excretion of phosphate with decreased osteocalcin levels (*p* = 0.16) in men. The other associations between calcium or phosphate excretion and bone turnover were not close to the statistical significance in the robust regression.

In women, uranium exposure was not associated with any indicators of bone turnover (Figure 2). Urinary excretion of neither calcium nor phosphate was associated with bone markers.

Those 32 study persons who reported a history of any bone fractures in adulthood were not statistically significantly more exposed to uranium than those without such history (median cumulative doses of uranium of 124 mg in those with fractures vs. 117 mg in those without). There were no differences in the levels of the markers of bone metabolism among those with or without past fractures (data not shown).

## Discussion

The uranium exposure covered a wide range of concentrations in this study. More than half of the study persons used drinking water with uranium concentration exceeding 15 μg/L, which is the new provisional World Health Organization (WHO) guideline value for uranium in drinking water (WHO 2004).

In men, chronic uranium exposure indicated by uranium level in drinking water as well as daily and cumulative uranium intakes tended to be associated with the increased levels of the bone resorption marker CTx and to a lesser degree of the bone formation marker osteocalcin. The association of uranium exposure and CTx reached a marginal significance at the 5% level in the robust analysis that down-weights the influence of possible outliers and was significant in the conventional regression analysis. This finding may indicate that bone is a possible target of chemical toxicity of natural uranium.

In contrast to men, no statistically significant associations with uranium exposure and the measured bone turnover markers were observed in women. In women, subtle effects may be masked by other strong determinants of bone turnover, such as menopause and hormone use. Potential confounding factors including menopausal status, recent body weight changes, physical activity, and calcium and vitamin D supplementation could not be effectively controlled in the present study, although they all have an influence on bone metabolism (Delmas 2000; Watts 1999).

The subtle effects of uranium on bone markers in men could be explained by different mechanisms. Accumulation of uranium in bone may have a local effect on bone metabolism or structure. Direct effect of uranium on bone has been shown in animal studies, with accumulation of uranium into bone (Leggett and Pellmar 2003; Pellmar et al. 1999). In laboratory animals exposure has been shown to modify bone formation and resorption (Diaz Sylvester et al. 2002; Guglielmotti et al. 1984; Ubios et al. 1991). Direct bone effect is also supported by the present observation that the change in bone markers was more strongly associated with uranium concentration in drinking water and daily or cumulative intake than with uranium concentration in urine. Concentration in water and intake likely describe long-term exposure to uranium and consequently accumulation to bone better than concentration in urine, which reflects recent exposure.

Uranium might have an effect on bone also via its influence on kidneys. Chronic renal insufficiency has been reported to affect bone metabolism (Malluche 1995; Malluche and Faugere 1990). On the other hand, elevated levels of bone markers have been observed in patients with kidney damage due to chronic exposure to cadmium, another metal toxic to the kidney (Aoshima et al. 2003; Kido et al. 1990). Cadmium-induced osteoporosis is associated specifically with tubular damage, including increased excretion of calcium in urine (Jin et al. 2004). However, uranium in drinking water does not cause severe kidney damage or cytotoxicity even at high exposure levels (Kurttio et al. 2002; Zamora et al. 1998), nor had the subjects in the present study significant kidney insufficiency. We have earlier shown that intake of natural uranium in drinking water is associated with increased fractional excretion of calcium and phosphate (Kurttio et al. 2002). By increasing leakage of calcium into urine by disturbing its tubular reabsorption, uranium exposure could secondarily lead to bone resorption. However, the increased excretion of calcium and phosphate in urine has been suggested to be associated most strongly with the recent uranium exposure (uranium in urine) (Kurttio et al. 2002), and in the present study CTx was associated with long-term exposure to uranium (concentration in water, and daily and cumulative intake). Accordingly, on the basis of earlier animal studies and the present results, we propose that the relationship between uranium intake and bone markers reflects the direct effect of uranium on bone.

Only uranium was analyzed from the water samples. It is possible that other elements or constituents in drinking water confound the results. To be a confounding factor, it should be associated with both uranium concentration and the outcome measures. However, other heavy metals, including cadmium and lead, occur extremely rarely in substantial concentrations in Finnish drilled wells and are not correlated with uranium concentrations (Kurttio et al., unpublished data). Therefore, other elements in drinking water are very unlikely to confound the results.

In this study population, drinking water is expected to be the predominant source of uranium, especially among those with elevated uranium concentrations in well water. The study persons had used drinking water from the drilled wells with measured uranium concentrations for at least 1 year. Therefore, a steady state uranium exposure can be anticipated.

Uranium concentration in the private wells drilled in bedrock may vary considerably over time, and therefore a spot sampling may not accurately represent the long-term uranium exposure. Additionally, the daily and cumulative intakes of uranium are based on study persons’ own estimates on their drinking water consumption, which also adds uncertainty. Although urinary uranium concentration is unaffected by these sources of uncertainty, it is limited mainly to current uranium exposure. Yet there is a high correlation between uranium exposure indicators.

Substantial variation in age complicates the interpretation of the results because several age-dependent factors influencing the bone turnover may mask possible effects of uranium. As was seen in this study, the levels of bone turnover markers remain approximately stable from 25 years of age to menopausal age (~ 55 years) in women and to 65 years of age in men. Obviously focusing on limited ages would facilitate the interpretation of the results.

## Conclusions

We found some evidence for an association between increased bone turnover and exposure to natural uranium through drinking water among men. The fact that similar effects were not observed in women may be due to other stronger factors in bone metabolism of women that may mask the effects of uranium. This study suggests that in addition to kidneys, bone may be another target for uranium toxicity.

## Figures and Tables

**Table 1:** Description of age and smoking by sex in the study population.

	Men [no. (%)]	Women [no. (%)]
Age (years)		
< 45	42 (29)	46 (32)
45–55	34 (23)	40 (28)
55–65	50 (34)	30 (21)
≥65	20 (14)	26 (18)
Smoking		
Never	69 (47)	94 (66)
Ex	53 (36)	31 (22)
Current	18 (12)	14 (10)
Missing	6 (4)	3 (2)
Total	146 (100)	142 (100)

**Table 2:** Basic information on the study population, uranium exposure, and levels of indicators of bone turnover and urinary calcium, phosphate, and creatinine.

				Percentile			
Characteristic	No.	Mean	Median	25th	75th	Minimum	Maximum
Men							
Age (years)	146	53	54	44	61	26	78
Body mass index (kg/m^2^)	143	26	25	24	28	20	35
Duration of the use of drilled well (years)	146	13	11	6	20	2	34
Uranium in drinking water (μg/L)	146	124	28	6	122	0.087	1,920
Daily intake of uranium from drinking water (μg)	146	216	36	8	207	0.2	4,128
Cumulative intake of uranium from drinking water (g)	146	1.33	0.12	0.02	0.60	0.001	33
Uranium in urine (μg/L)	146	0.29	0.06	0.01	0.27	0.001	4.54
Uranium in urine (μg/mmol creatinine)	146	0.041	0.007	0.002	0.032	0.0001	0.333
Urine calcium (mmol/hr)	146	0.7	0.3	0.1	0.6	0.04	19
Urine phosphate (mmol/hr)	146	3.9	2.6	1.3	4.6	0.3	19
Urine creatinine (mmol/L)	146	8.5	7.8	5.4	10.4	1.2	28
Serum osteocalcin (μg/L)	146	21	20	16	25	7	54
Serum P1NP (μg/L)	146	42	37	31	48	15	178
Serum CTx (nmol/L)	146	3.4	2.4	1.7	3.3	0.4	65
Women							
Age (years)	142	52	53	43	61	28	83
Body mass index (kg/m^2^)	128	25	24	22	26	18	41
No. of deliveries	82	2	2	2	3	0	6
Duration of the use of drilled well (years)	142	13	11	6	19	1	34
Uranium in drinking water (μg/L)	142	113	26	5	115	0.001	930
Daily intake of uranium from drinking water (μg)	142	212	36	7	207	0.0	2,748
Cumulative intake of uranium from drinking water (g)	142	1.21	0.12	0.03	0.73	0.000	30
Uranium in urine (μg/L)	142	0.38	0.09	0.02	0.42	0.001	3.25
Uranium in urine (μg/mmol creatinine)	142	0.075	0.019	0.004	0.087	0.0003	0.571
Urine calcium (mmol/hr)	141	0.4	0.2	0.1	0.5	0.03	3.0
Urine phosphate (mmol/hr)	141	3.0	1.7	1.0	3.3	0.1	17
Urine creatinine (mmol/L)	142	6.3	5.4	3.7	7.8	0.9	24
Serum osteocalcin (μg/L)	142	21	19	15	24	6	121
Serum P1NP (μg/L)	142	38	34	26	47	9	152
Serum CTx (nmol/L)	142	2.8	2.3	1.5	3.3	0.4	40

**Table 3:** Correlation matrix for the log-transformed uranium (Ln U) exposure variables.

	Ln U in water (μg/L)	Ln U intake (μg/day)	Ln U cumulative intake (g)	Ln U in urine (μg/L)
Ln U in water (μg/L)	1			
Ln U intake (μg/day)	0.98	1		
Ln U cumulative intake (g)	0.93	0.95	1	
Ln U in urine (μg/L)	0.89	0.89	0.84	1
Ln U in urine (μg/mmol creatinine)	0.86	0.88	0.84	0.96

**Table 4:** *R*^2^ and *p*-values for the robust regression models of men and women including uranium concentration in water adjusted for age and smoking and estrogen use (for women).

	Men		Women	
Outcome	*R*^2^	*p*-Value	*R*^2^	*p*-Value
Osteocalcin	0.21	< 0.001	0.10	0.02
P1NP	0.13	< 0.001	0.12	0.008
CTx	0.14	< 0.001	0.12	0.00
